# Virophages of Giant Viruses: An Update at Eleven

**DOI:** 10.3390/v11080733

**Published:** 2019-08-08

**Authors:** Said Mougari, Dehia Sahmi-Bounsiar, Anthony Levasseur, Philippe Colson, Bernard La Scola

**Affiliations:** 1 Aix-Marseille Université, Institut de Recherche pour le Développement (IRD), Assistance Publique - Hôpitaux de Marseille (AP-HM), Microbes Evolution Phylogeny and Infections (MEPHI), 27 boulevard Jean Moulin, 13005 Marseille, France; 2 Institut Hospitalo-Universitaire (IHU) Méditerranée Infection, 19-21 boulevard Jean Moulin, 13005 Marseille, France

**Keywords:** virophage, giant virus, coculture, metagenomic, host-defense systems, satellite virus

## Abstract

The last decade has been marked by two eminent discoveries that have changed our perception of the virology field: The discovery of giant viruses and a distinct new class of viral agents that parasitize their viral factories, the virophages. Coculture and metagenomics have actively contributed to the expansion of the virophage family by isolating dozens of new members. This increase in the body of data on virophage not only revealed the diversity of the virophage group, but also the relevant ecological impact of these small viruses and their potential role in the dynamics of the microbial network. In addition, the isolation of virophages has led us to discover previously unknown features displayed by their host viruses and cells. In this review, we present an update of all the knowledge on the isolation, biology, genomics, and morphological features of the virophages, a decade after the discovery of their first member, the Sputnik virophage. We discuss their parasitic lifestyle as *bona fide* viruses of the giant virus factories, genetic parasites of their genomes, and then their role as a key component or target for some host defense mechanisms during the tripartite virophage–giant virus–host cell interaction. We also present the latest advances regarding their origin, classification, and definition that have been widely discussed.

## 1. Introduction

Viruses are the most abundant biological entities in the biosphere [1,2,3]. This virosphere encompasses a complex variety of billions of viruses that differ from each other according to their genome architecture and size, their virion structure, and their strategies for genome expression and replication [1,4,5,6]. These heterogeneous viruses infect almost all cellular life forms to propagate, including bacteria, archaea, and eukaryotes [7]. Perhaps one of the most astonishing replication strategies is to rely on the presence of another virus that infects the same host cell to replicate. This concept is that of satellite viruses and its discovery dates back to 1961 [8].

Giant viruses were discovered in 2003, and since then, they have sparked sustained interest [9,10]. They appear to be the most complex of the known viruses based on genomic and structural analyses [11,12,13,14,15,16,17]. They produce, inside the cytoplasm of their host cell, a complex viral factory that resembles a eukaryotic nucleus [18,19,20,21]. In addition, compared to some bacteria, giant viruses have larger virions and genomes that encode a similar or greater number of predicted genes [22,23].

Acanthamoeba polyphaga Mimivirus (APMV) was the first giant virus discovered [9]. It has several structural and genomic features that had not been described in viruses before its isolation [24]. The APMV capsid size is 500 nm with a dense layer of fibrils that can reach 140 nm in length [25,26,27]. The genome of APMV is, likewise, unique among viruses, being a double-stranded DNA of 1.2 megabase pairs (Mbp) encoding 979 putative proteins [11,28]. In 2005, APMV founded the family *Mimiviridae*, a new taxonomic group created based on its outstanding features [29].

In 2008, giant viruses were shown to be themselves the prey of another group of viruses that were named virophages [30]. Unlike satellite viruses, virophages were the first viruses that truly infected other viruses [31]. This feature has challenged the definition of a virus [24,32,33]. Virophages parasitize the viral factory of giant viruses, supposedly by hijacking the transcription and replication machinery of their virus host to express and replicate their own genomes [34]. Moreover, some virophages appear as *bona fide* parasites that negatively interfere with their host virus, inducing partial inhibition of reproduction of the giant virus and possibly increasing the genesis of defective particles [30,35,36].

Virophages, giant viruses, and their host cells seem to co-evolve with each other [37]. This relationship becomes increasingly obvious with the discovery of several virophage sequences integrated into giant viruses and host cells genomes [38,39]. Endogenized virophage sequences appear to play an instrumental role during the tripartite host cell–giant virus–virophage interactions, mainly as being involved in different host defense strategies [40,41,42].

In this review, we present the recent advances in the isolation and description of virophages, as well as the latest discoveries regarding their origin and classification, a decade after their discovery. We also discuss the role of virophages as invaders, preys, or antiviral weapons during the giant virus–host cell interactions.

## 2. Giant Virus Discovery

Virophages have been defined as obligatory parasites of giant virus factories and, therefore, their description was subsequent to the isolation of their viral hosts.

Since the discovery of the first giant virus APMV in 2003, dozens of other mimiviruses have been isolated by the same co-culture strategy using a panel of amoebas as biotopes and more recently by high-throughput systems based on flow cytometry and fluorescence staining [43,44,45,46,47]. Mimiviruses are currently composed of a wide variety of viruses isolated from various ecosystems from all five continents [15,48,49,50,51,52]. Based on the conserved genes, such as family B DNA polymerase encoding gene sequences as well as phylogenomic analyses, mimiviruses of amoebae were found primarily to belong to three lineages, designated A, B, and C: APMV is the pioneer member of lineage A, while Moumouvirus and Megavirus chiliensis are the prototype members of lineages B and C, respectively [53,54].

Since 2010, other giant viruses, distantly linked to mimiviruses, have been discovered. They include Cafeteria roenbergensis virus (CroV) that infects the marine phagotrophic biflagellate *C. roenbergensis* [12], Phaeocystis globosa virus that infects the unicellular marine protists *Phaeocystis globose,* and other distant mimiviruses [52,55,56,57,58,59,60,61,62,63,64,65,66,67,68,69].

Co-culture on amoebae also led to the isolation from diversified environmental and biological samples of dozens of other giant viruses that differ from mimiviruses. Some of these newly discovered giant viruses founded a new viral family named *Marseilleviridae,* with Marseillevirus as pioneer representative [70,71]. Others are not yet officially classified by the international committee of taxonomy of viruses (ICTV): They include pandoraviruses, pithoviruses, faustoviruses, Mollivirus, cedratviruses, Kaumoebavirus, pacmanviruses, and Orpheovirus [72,73,74,75,76,77,78,79], in addition to other putative giant virus genomes assembled from metagenomes [57]. All these new viral families and putative groups were linked to the nucleo-cytoplasmic large DNA viruses (NCLDV), a previously defined monophyletic group of viruses that infect animals and diverse unicellular eukaryotes [80,81,82]. The NCLDVs share a major biological feature—the cytoplasm of their host cells contains specific structures known as “viral factories” that are the sites of the viral genome replication and the viral particle morphogenesis [17]. According to their phylogenetic, structural, and biological features, the five predefined families of NCLDV and the families *Mimiviridae* and *Marseilleviridae* were proposed in 2013 to be reclassified into a new proposed viral order, Megavirales [83].

## 3. Virophage Discovery over a Decade

### 3.1. Virophages Isolated by Culture

#### 3.1.1. Sputnik, the First Virophage

**Figure 1:**
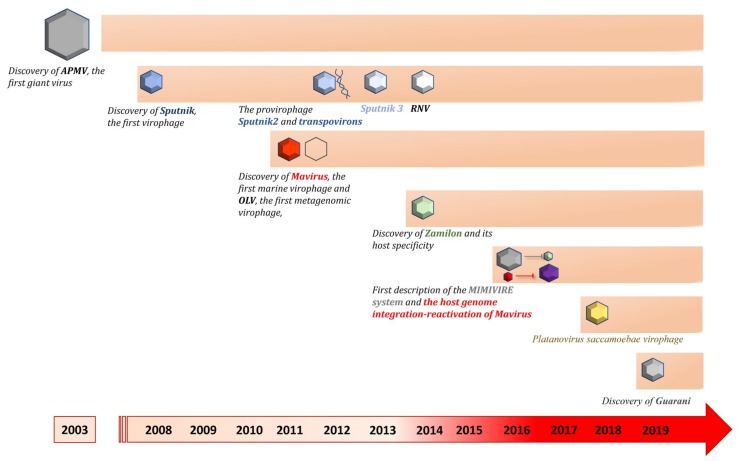
Timeline showing the chronological order of description of virophages isolated by co-culture and the major discoveries in the virophage field. RNV: Rio Negro Virophage. OLV: Organic Lake Virophage.

The study of mimiviruses has paved the way for the isolation of a new class of viral agents that depend on the infection of their host cells with a mimivirus to replicate. This new viral entity was named virophage according to its typical lifestyle, by analogy to that of the bacteriophage. The discovery of virophages dates back to the isolation of the second giant virus of amoebae in 2008, a second mimivirus strain and close APMV relative, Acanthamoeba castellanii mamavirus (Figure 1) [30]. The Mamavirus factory was colonized by small virions about 50 nm in diameter that impaired its infectivity, resulting in a decrease in amoebae lysis. In addition, the morphogenesis of the mimivirus appeared to be impaired with a high rate of production of abnormal particles. This new small virus was baptized Sputnik virophage [30]. Sputnik has an 18,343 bp-long double-stranded DNA genome that harbors 21 putative genes (Table 1). These genes encode major and minor capsid proteins (MCP and mCP, respectively) and proteins predicted to be involved in DNA replication [84]. Genomic and structural study of the virophage showed that the MCP, encoded by the V20 gene, contains 595 amino acids, which is in the same magnitude range as for Mimivirus MCP (437 amino acids). In addition, CryoEM analysis revealed that, like for Mimivirus, the MCP of Sputnik has a double jelly-roll fold [85,86]. However, the Sputnik MCP was not homologous, neither to the Mimivirus MCP, nor to any other sequences present in databases at the time the Sputnik virophage was isolated. This suggests that Sputnik evolved from other mobile genetic elements before its association with Mimivirus [85,86].

#### 3.1.2. Other Virophages of the Sputnik Clade

**Figure 2:**
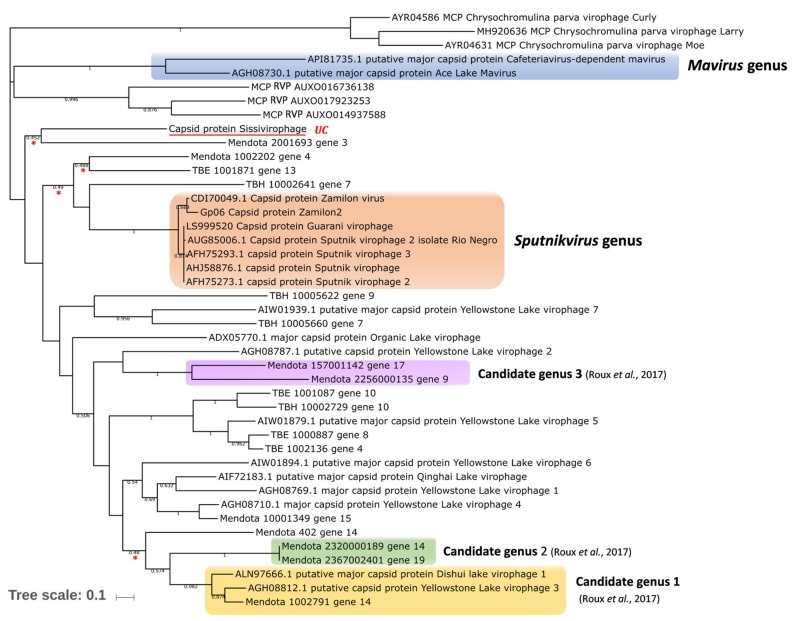
Phylogenetic reconstructions based on the capsid proteins of known virophages with their current/proposed taxonomy. The analysis was performed using MEGA version 7.0, applying the maximum-likelihood method and WAG ((Whelan & Goldman) model of evolution with 500 bootstrap replicates. Cutoff ≥ 50%, branches with 45%–50% support are marked with a red asterisk. UC: Under characterization.

Following the discovery of the Sputnik virophage, other virophages, very similar to Sputnik, were isolated. Sputnik 2 was isolated in association with Lentille virus, a mimivirus from the lineage A that has been cultivated from a contact lens rinsing liquid of a patient with keratitis (Table 1). Sputnik 2 was integrated as a provirophage into this mimivirus genome [38,93]. Unexpectedly, a new class of mobile genetic elements, that have been named transpovirons, was also discovered in the Lentillevirus genome (Figure 1) [38]. In contrast, the third Sputnik strain was found without its natural host mimivirus in a soil sample collected in Marseille, France (Table 1 and Figure 1 and Figure 2). Indeed, the culture of Sputnik 3 was performed using a new protocol based on the use of a helper mimivirus experimentally introduced in the biotope to propagate the virophage [88]. Rio negro virophage (RNV), the fourth Sputnik strain, was isolated from the Negro River in Amazonia, Brazil (Table 1 and Figure 1 and Figure 2). A giant virus from the *Mimivirus* genus was isolated from the same sample. This mimivirus belongs to lineage A and has been named Sambavirus [94]. The sizes of Sputnik 2, 3, and RNV particles are similar (50–74 nm) to that of the Sputnik prototype isolate. The genomes of these virophages are also highly similar to that of Sputnik. Sputnik 2 and 3 have an 18,338 bp double-stranded DNA genome encoding putatively 20 genes [30,35,38,88]. Likewise, the genome of RNV is 18,145 bp in length and encodes 20 genes [90]. Three genes from the Sputnik strains are closely related to the mimivirus genes, whereas most of the other genes encode proteins of unknown origin and function (13 ORFans). The remaining genes have homologs in bacteria and archaea [35].

The Guarani is a Sputnik-like virophage isolated in 2019 from a water sample collected in Pampulha lagoon, Belo Horizonte, Brazil (Table 1 and Figure 1 and Figure 2). Like Sputnik 3, the virophage was found free of its natural giant virus host. Therefore, a helper mimivirus (APMV) has been used to propagate the virophage. Guarani has an 18,967 bp-long double-stranded DNA genome that encodes 22 predicted genes and is very similar to Sputnik genomes. It differs from Sputnik’s genomes in two genes, ORF19 and ORF12. A distant homologue of ORF19 was found in Zamilon, but ORF12 has no match with any sequence and its G+C content appears to be different from background G+C content of the rest of Guarani genome. Another interesting feature observed in Guarani is the timing of its replication cycle compared to that of its giant viral host. Indeed, all previously isolated virophages were suspected of having a late replication cycle due to the presence of the late promoter of Mimivirus upstream of most of their genes. For Guarani, the late replication has been experimentally confirmed by quantifying the virophage DNA across the replication cycle. In addition, the authors were unable to observe the virophage before a late phase of the replication cycle. These results have been confirmed by the detection of the Mimivirus late promoter upstream of 10 Guarani genes, including genes encoding for proteins related to DNA replication and morphogenesis [92].

#### 3.1.3. Mavirus Virophage

This virophage was named Mavirus because of its genetic homology to Maverick or Polintons, a large class of self-replicating eukaryotic transposable elements (Mavirus means Maverick-like virus). Mavirus is a marine virophage that infects the giant Cafeteria roenbergensis virus (CroV), a distant Mimivirus relative [12,87]. Their host cell is the marine phagotrophic flagellate *Cafeteria roenbergensis* that belongs to the *Chromalveolata* phylum. The comparison of the genome of CroV with those of giant viruses from the *Mimiviridae* family led to its classification as a member of this family [95]. The size of the Mavirus capsid and genome are very similar to those for Sputnik (60 nm and 19,063 bp, respectively). The genome encodes 20 putative genes (Table 1) [87]. This virophage inhibits the replication of its host virus CroV and thus increases the survival of the *Cafeteria roenbergensis* cells [40]. Interestingly, Mavirus genome analysis has provided a support to the existence of an evolutionary relationship between this virophage and Polintons. Indeed, Mavirus and Polintons share seven homologous coding sequences, including those encoding the protein-primed DNA polymerase B and the rve-superfamily retroviral integrase (PolB and rve-INT, respectively), which are highly conserved in Polintons. According to the similarities in genome length and architecture between Mavirus and Polintons, it has been proposed that Polintons evolved from a virophage ancestor [87]. However, this hypothesis remained controversial and an alternative scenario was proposed to explain the evolutionary relationship between virophages and Polintons, according to which virophages evolved from Polintons and not the opposite (see below in part: Virophages in the context of evolution).

#### 3.1.4. Zamilon Virophage

The Zamilon virophage was isolated in 2014 from a soil sample collected in Tunisia (Table 1 and Figure 1 and Figure 2). In contrast to Sputnik that is able to decrease the infectivity and impact the morphogenesis of its giant virus host, Zamilon does not seem to have any effect against its host virus replication (hence the name Zamilon, which means “colleague” in Arabic) [89]. One would suppose that this propriety may question the concept of virophage for this virus. Further tests are required to characterize its impact on other mimiviruses or distant relatives. The second interesting feature of Zamilon is its host specificity. Indeed, it has been demonstrated that Zamilon is the unique virophage able to replicate within factories of mimiviruses from lineages B and C but not within those from lineage A [35,89]. The genetic basis of this host specificity has recently been studied and has suggested an arms race between giant viruses and their small parasites (see below in part: MIMIVIRE, a giant virus defense system) [41]. On the other hand, the genome of Zamilon is a 17,276 bp double-stranded DNA genome, which contains 20 genes. This genome is close to that of Sputnik (75% identity and 76% coverage with Sputnik genome). Similarly, most of the Zamilon genes present a moderate-to-high similarity of identity with Sputnik genes (from 31%–86% identity) [89].

#### 3.1.5. Virophages Isolated by Culture Partially Characterized

Platanovirus saccamoebae is the proposed name of a Mimivirus-like giant virus that has been isolated from a sycamore tree using *Saccamoeba lacustris* as cell support [96]. Surprisingly, this giant virus has been associated with small virions with a diameter of 50–60 nm that replicated inside its viral factory [91]. These viruses appeared to have a negative impact on the giant virus replication. Genome analyses of the giant virus revealed the greatest similarity of identity with the Megavirus chiliensis genome, but there are no available data for the virophage genome (Table 1 and Figure 1).

We recently isolated a new virophage that we named Sissivirophage (data not published) [52]. Although this virophage has not yet been fully characterized, we were able to sequence its genome and visualize the morphology of its particles. We found that its genome was very divergent from the other known isolated virophages. Indeed, the phylogenetic tree based on Sissivirophage MCP (GenBank: MN151334) shows that the closest homolog is from a virophage assembled from metagenomic data from Lake Mendota, USA (see below). Further experiments and analyses are ongoing to characterize the genomic, biological, and ultrastructural features of this new member of the virophage group (Figure 2).

In addition to these virophages, a second strain of Zamilon (Zamilon virus isolate Z.vigne ) was isolated in France, and sequenced by Jeudy et al.; while the characterization of the virophage has not yet been published, this virophage has a 17,327 bp double-stranded DNA genome encoding 20 predicted genes (GenBank: MG807318.2).

### 3.2. Virophages Discovered by Genomic

Phaeocystis globosa virus virophage (PgVV) is a virophage that has been assembled during genome sequencing of a phycodnavirus named PgV-16T [62]. This giant virus was isolated from a Dutch coastal water sample. The virophage has a 19,527 bp-long double-stranded DNA genome encoding 16 predicted ORFs (Table 2 and Figure 2). Ten of them have no functional annotation (hypothetical proteins). Two PgVV genes encode for a predicted endonuclease and DNA primase/polymerase, which are homologs to two Mavirus genes (ORF2 and ORF4, respectively in PgVV), in addition to a gene similar to ORF1 of OLV. PgVV was also related to Polintons and, thus, it is a member of a new group of viruses known as Polintons-like-viruses (PLV) [97]. No virophage virions were observed in infected *Phaeocystis globosa* culture cells. Therefore, it has been proposed that the virophage may be carried in the giant virus genome as a linear plasmid or a provirophage, as for the case of Sputnik 2 in the Lentille virus genome [62].

In 2019, a new distant Mimivirus relative named CpV-BQ2 that infects *Chrysochromulina parva* was isolated from Lake Ontario in North America. Three putative virophage genomes, most closely related to the PgVV, were assembled during genome sequencing of CpV-BQ2. The new virophages were named CpV-PLV Larry, CpV-PLV Curly, and CpV-PLV Moe (Figure 2). Their genomes are approximatively 22 kbp in length and encode between 19 and 23 predicted genes (Table 2). No virophage particles were observed in the samples from which the virophage sequences were obtained. It has been suggested that these virophages could exist as provirophages or were encapsidated within the CpV-BQ2 virions [69].

### 3.3. Virophages from Metagenomic Datasets

Although the first virophage was reported in 2008, 11 years ago, coculture strategies contributed to isolate only ten strains of virophage (described above). In contrast, 57 novel complete or partial virophage genomes were reported from different metagenomic datasets [98,99]. Metagenome assemblies not only revealed the diversity of the virophage group, but also suggested a relevant ecological impact of these small viruses and their potential role in the stability of the microbial network [55,98].

The first virophage detected by metagenomics was the Organic Lake virophage (OLV). OLV is also the second virophage discovered after Sputnik. Its genome was assembled by metaproteogenomic analyses using samples collected from the Organic Lake, a hypersaline meromictic lake in Antarctica [55]. OLV has a 26,421 bp double-stranded DNA genome that encodes 26 putative genes (Table 2 and Figure 1 and Figure 2). Several OLV genes have homologs in the Sputnik genome with approximatively 27%–42% amino acid identity. In addition, Sputnik-like particles of 50 nm in diameter were observed by transmission electron microscopy in the same sample from which the virophage genome was detected. Furthermore, a nearly complete genome from a putative giant virus, primarily identified as a phycodnavirus then reclassified as distant Mimivirus relative, was also reconstructed from the same metagenome. This giant virus was named Organic Lake phycodnavirus (OLPV) and probably represents the giant virus host of OLV.

Metagenome assembly for samples from the Yellowstone Lake (USA) allowed the construction of seven genomes of virophages, which were named Yellowstone Lake virophages (YSLVs) [100,101]. Their genome sizes range from 23–29 kbp with 21–34 predicted genes. Ace Lake Mavirus (ALM) is a virophage whose near-complete genome has been assembled from the Antarctica Aquatic Microbial metagenome in 2013. It is a 17,767 bp-long double-stranded DNA encoding 22 predicted ORFs (Table 2 and Figure 2). Interestingly, 14 ALM genes have homologs in the Mavirus genome (hence the name Ace Lake Mavirus) [101].

In 2015, 16 MCP virophage sequences were identified from distinct metagenomes including those generated from activated sludge, freshwater sediment, bioreactor, marine water, wastewater, and sheep rumen. In addition, two near-complete and one partial virophage genomes were assembled from a rumen metagenome and were named RVPs (Table 2 and Figure 2) [102]. The rumen virophage genomes are linear and two of them contain terminal inverted repeats. Interestingly, the RVPs appear to be virophage–Polintons hybrids, because they have capsid proteins related to those of virophages, while their B family polymerase (PolB) encoding genes are closer to Polintons. Moreover, sequences similar to the MCP of mimiviruses, which probably represent the giant virus hosts, have been identified in most of the metagenomes from which the virophages sequences were obtained.

In parallel, a partial genome of a Zamilon virophage designated Zamilon 2 (6716 bp) was assembled from the same metagenome of the bioreactor explored in the previous study (Table 2 and Figure 2) [99]. This genome comprises 15 predicted ORFs with 78% to 99% similarity of identity (in amino acids) to Zamilon virophage genes that include genes encoding the capsid protein and DNA replication and DNA packaging proteins.

In 2016, the genome of Dishui Lake virophage (DSLV) was discovered in the Dishui Lake (China) [103]. PCR and metagenomic analyses have shown that its length is 28,788 bp and that it harbors 28 putative genes, most of which have significant similarities with homologs of other known virophages (Table 2 and Figure 2). In addition, 46 other virophage sequences, including from 6 genes related to MCPs closely related to homologs from OLV and YSLVs, were detected in the same sample.

The same year, Qinghai Lake virophage (QLV) was identified in the Qinghai Lake (Tibetan mountain) and its genome was proposed to be circular, 23,379 bp in length, and with 25 predicted ORFs (Table 2 and Figure 2) [104]. Eleven of the QLV genes are specific to this virophage as they have not been detected in the known virophages. However, other genes have distant homologs in other virophages, including YLSVs, OLV, Sputnik, Zamilon, and Mavirus.

More recently, metagenomic analyses performed at different time points on samples collected from two American freshwater lakes (Trout Bog Lake and Lake Mendota in the USA) revealed the presence of sequences from 25 different virophages [98]. Seventeen near-complete or complete virophage genomes were assembled. These putative complete genomes ranged from 13.8 to 25.8 kbp in size and contain from 13 to 25 predicted ORFs (Table 2, Figure 2). In addition, phylogenies reconstructed based on single and concatenated marker genes revealed that the freshwater virophages are divergent from the two established virophage genera, which are the genus *Sputnikvirus* and the genus *Mavirus* [105]. Therefore, three new candidate virophage genera have been proposed in the family *Lavidaviridae* in order to accommodate the newly discovered putative virophages.

The presence of 19 virophage sequences was also correlated with that of at least one NCLDV member, which probably represents the giant virus host. Based on the PolB gene and other signature genes, these putative giant viruses, whose sequences co-occurred with those of the putative virophages, were affiliated with families *Mimiviridae* and *Phycodnaviridae*.

## 4. Virophages in the Context of Evolution

Diverse eukaryotic genomes harbor various classes of integrated large transposable elements known as Polintons or Mavericks [106,107]. All Polintons share two key enzymes implicated in their transposable lifestyle that are a protein-primed type B DNA polymerase and an integrase [106,107]. Most Polintons encode a packaging ATPase, a C5-family protease, and two capsid proteins with double jelly-roll fold, MCP and mCP. The presence of viral morphogenesis genes suggested that some of these mobile genetic elements could produce virions (polintonviruses) under specific conditions and could therefore have a dual lifestyle by combining features of viruses and transposable elements [108]. This finding raised questions about a dramatic evolutionary relationship between bacteriophages, Polintons, virophages, and giant DNA viruses of the proposed order Megavirales. It has been proposed that polintonviruses were the first viral entities that evolved from bacteriophages and then became the ancestors of most DNA viruses of eukaryotes, comprising virophages and giant viruses [6,109,110]. Further, phylogenomic analyses of the genomes of the three virophages Sputnik, Mavirus, and Organic Lake virophages revealed that all these viruses share six homologous proteins, including Primase Superfamily 3 helicase, packaging ATPase, Cysteine protease, Zn-ribbon domain containing protein, MCP, and mCP [109]. These two latter virion proteins have no known homologs outside the virophages, suggesting that all virophages have evolved from a common ancestor. Surprisingly, two of the other four core virophage genes, packaging ATPase and maturation protease, are present in Polintons. Furthermore, the Mavirus virophage share with Polintons the PolB and the integrase genes in addition to the morphogenesis coding elements. The phylogenetic trees based on these two conserved proteins show that Mavirus branches within the clade of Polintons. In addition, the phylogenetic tree based on the Cysteine protease domain shows that Mavirus and two other virophages, Sputnik and OLV, are clustered together and with the Polinton clade [109].

Recently, Yutin et al. discovered a new family of virophages that have chimeric genomes in a rumen metagenome [102]. In these genomes, MCP is homologous to virophages MCP, whereas PolB seems closely related to polinton homologs. Therefore, it has been proposed that the genome of these virophages evolved through a recombination between a viral form of Polintons and a virophage ancestor that co-infected an amoeba infected with a mimivirus. Another scenario is that Polintons evolved from viruses (and not *vice versa*) and that virophages are not descendants from Polintons but share the same ancestor virus whose nature and identity are undetermined [111].

## 5. The Virophage Lifestyle

### 5.1. Replication Cycle

#### 5.1.1. Entry

**Figure 3:**
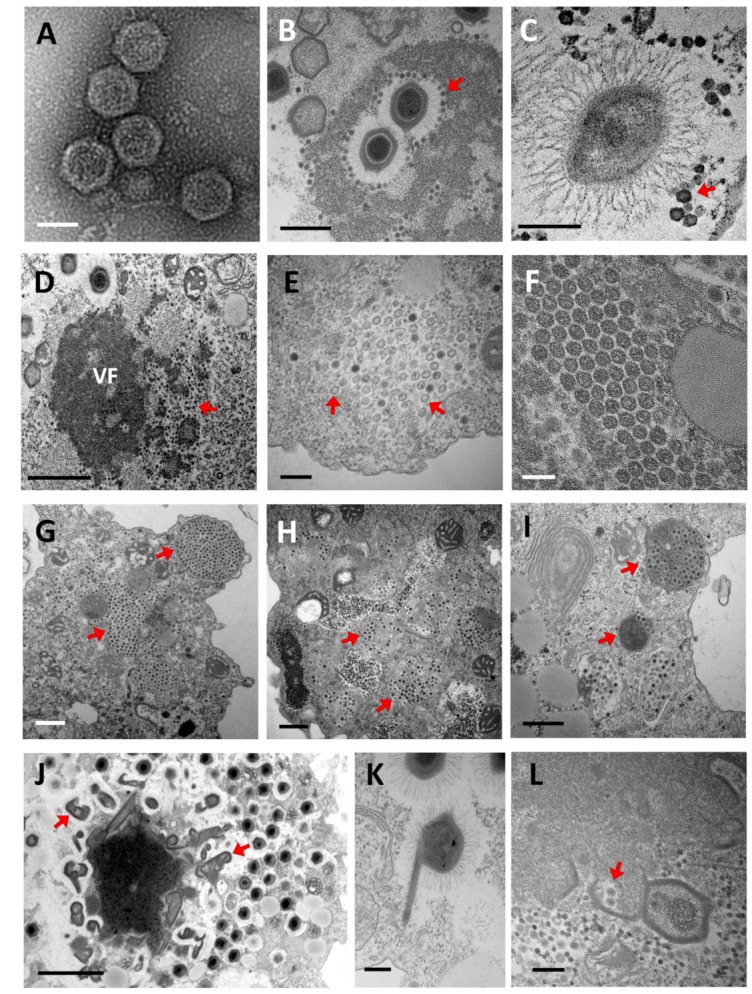
Electronic microscopy observation of virophage particles and their replication cycle. (**A**) Negative staining electronic microscopy observation. (**B**–**L**) Transmission electronic microscopy images. (A) Morphology of purified Sputnik virophage particles (scale bar, 500 nm). (**B,C**) Sputnik and Guarani virions attached to the Mimivirus fibrils, respectively. (Scale bar, 500 nm and 200 nm). (**D**) Sputnik virophage invading the virus factory of acanthamoeba polyphaga Mimivirus (APMV) (scale bar, 1 µm). (**E**) Immature Sputnik virions observed in the cytoplasm of *A. castellanii* during co-infection with APMV (arrows) (scale bar, 200 nm). (**F**) Mature Sputnik virions (scale bar, 100 nm). (**G**–**I**) Virophages virions are commonly observed clustered inside typical cytoplasmic vesicles at the end of their replication cycles (arrows). (G) Sputnik progeny. (**H**) Zamilon progeny. (I) Guarani progeny (scale bars, 500 nm). (**J**,**K**) The genesis of abnormal Mimivirus particles has been observed during infection with virophages (arrows). (scale bars, 2 µm and 200 nm). (L) Encapsidation of virophage virions within the Mimivirus capsid (arrows) (scale bar, 200 nm). VF: Virus factory.

Two different modes of entry have been proposed [112]. Sputnik strains are supposed to use the paired-entry mode where they infect the host cell simultaneously with their giant virus host, attaching to its capsid fibrils. This model gets support from multiple electronic microscopy images showing Sputnik progeny trapped in mimiviruses fibrils (Figure 3A,B) [112,113]. It has been hypothesized that the mushroom-like fibers that coat the virophage capsid interact with the peptidoglycan-like structure that covers the mimivirus fibrils to allow association between the virus-virophage particles [85,113]. In addition, Mimivirus lacking fibrils were found to be resistant to Sputnik infection, thus reinforcing this hypothesis [114]. The second mode of entry of virophages has only been observed with Mavirus, which infects its host cell independently of the giant virus (CroV) through clathrin-mediated endocytosis, as observed by electronic microscopy [87,112].

#### 5.1.2. Genome Release, Expression, Replication, and Viral Morphogenesis

After entry, the next step is characterized by the release of the capsid content, notably the genome. The structure of the Sputnik particle shows that its capsid is composed of 260 trimeric capsomers and 12 pentameric capsomers assembled into a T = 27 lattice [85,86]. It has been proposed that the Sputnik genome delivery occurs after a loss of one or more pentameric capsomers, most likely induced by such a stress as pH reduction [85,86]. The virion content that is released comprises an arsenal of ready-to-use transcripts from all but one of the virophage genes [113]. Although the function of these packaged mRNAs remains unknown, they could be used at the early stage of infection, immediately after the virus uncoating, to initiate the parasitic pathway of Sputnik with its giant virus [113].

Virophages are defined as *bona fide* parasites of giant viruses. Therefore, they are deemed to depend on the transcription and DNA replication machinery of their so-called virus hosts, rather than on that of the cell host. Indeed, the mimivirus promoter associated with late expression was found upstream of 12 Sputnik genes, while the conserved CroV late promoter was detected upstream of all Mavirus genes [87,115]. In addition, a hairpin polyadenylation signal specific to Mimivirus and absent from amoeba transcripts was detected at the end of 16 Sputnik genes [34]. While it has been proposed that Mavirus genome replication was governed by the virophage-encoded replication machinery as this virophage encodes its own DNA polymerase [116], no RNA or DNA polymerase genes were identified among the 21 Sputnik genes [115]. Late replication of genetic material and virions have been experimentally demonstrated for Guarani. This suggested that Sputnik gene expression is catalyzed by the transcription–replication complex of the giant virus [31,34,113]. These replication steps probably occur inside the giant virus factory, according to electron microscopy and immunofluorescence images showing Sputnik viruses produced from this site (Figure 3D) [113]. At the end of the replication cycle, four virophage-encoded proteins, including MCP, mCP, the ATPase, and the cysteine protease, are thought to be involved in the virion assembly and maturation [117].

MCPs and mCPs assemble into virion-like icosahedral particles. The shape of the virophage progeny appears to be provided by the mCPs that correspond to penton associated proteins [117]. This assembly step does not seem to require the presence of a specific giant virus or cellular initiation factor. After the assembly, it has been proposed that the packaging ATPase packages the virophage genome inside the virion, and then the cysteine protease processes the MCPs at their C-terminal part. This seems to increase the stability of the capsid at low pH and prepares virions for their maturation by detaching the double-stranded DNA genome from the capsid inner surface prior to its release during the next replication cycle [117].

### 5.2. The Impact of Virophage Infection on the Giant Virus Cycle

It has been shown that most virophages induce a significant negative effect on the replication cycle of their giant virus host. Sputnik, Mavirus, and RNV have been involved in a drastic decrease of their host giant virus propagation resulting in an increase in the host-cell population survival [30,87,88,94]. Moreover, while defective particles can form even in the absence of virophages, Sputnik causes a highly detrimental impact on the viral host morphogenesis, leading to an increase in the formation of diseased particles (Figure 3J–L) [30,118]. Otherwise, co-infection with Sputnik and Marseillevirus affected the capacity of Marseillevirus to replicate by delaying the appearance of its virus factory without increasing the Sputnik titers [113]. Recently, a new virophage has been isolated with a Mimivirus-like giant virus that infects *Saccamoeba* spp. This virophage appears to be deleterious to the giant virus by inducing a 70% reduction in viral capsid production, which has also decreased by three times amoeba lysis. [91]. Guarani virophage also causes a significant decrease in the host giant virus replication without clear alteration of the viral host morphogenesis [92]. In contrast, co-infection with Zamilon neither reduced the capacity of the giant virus to lyse the host amoeba nor altered the giant virus replication or morphogenesis [89].

**Figure 4:**
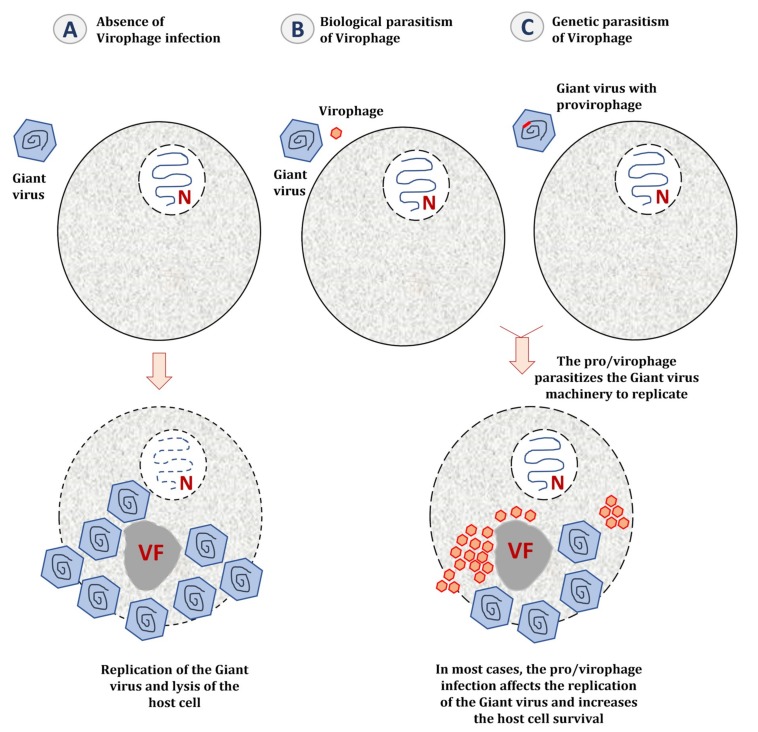
The parasitic lifestyle of virophages. (**A**) When the host cell is only infected by a giant virus, the latter establishes a cytoplasmic virus factory to replicate and generates new virions, and the host cell is most likely lysed at the end of its replication cycle. (**B**) When the host cell is co-infected with a giant virus and its virophage, the latter parasitizes the giant virus factory. The presence of virophages could seriously impact the infectivity of the giant virus by decreasing its replication efficiency and increasing the survival of the host cell. (**C**) When the giant virus genome is parasitized by a provirophage, the latter is expressed during the giant virus replication. The virophage is produced from the giant virus factory and inhibits the giant virus replication, thus increasing the host cell survival. VF: Virus factory.

In most cases, the presence of a virophage seems to be instrumental in the giant virus–host cell interaction (Figure 4) [119]. By decreasing the virulence of giant viruses, virophages regulate the dynamics of the cellular population (amoebae and marine protists) [120]. A metagenomic study performed on samples from Organic Lake, a hypersaline meromictic lake in Antarctica, suggested that virophages stimulate the growth of phototrophic algae by reducing their mortality caused by the giant viruses [55]. In addition to this ecological study, a mathematical model was applied to study the dynamic interaction between giant viruses, virophages, and cellular hosts. In this model, the virophages not only protect their host cells directly by interfering with the replication of their giant virus, but also indirectly by selecting viral clones with weaker reproductive ratio [121].

## 6. Virophages as Mobile Genetic Elements of Giant Viruses and Their Host Cells

### 6.1. Genetic Parasitism of the Giant Virus Genome

Virophages target the virus factory and the replication machinery of their host and also integrate their own genome in that of their viral hosts (Figure 4). It has been demonstrated that Sputnik 2 can target and integrate approximately any region in the genome of Lentille virus [38,93]. The infection of amoebae with Lentille virus is associated with an active expression of the provirophage and thus, a production of Sputnik 2 particles from the mimivirus factory (Figure 4). Provirophage may be the ultimate stage of parasitism developed by virophages to promote their expansion in the biosphere by being an integral part of their viral host particle.

### 6.2. Integration of Virophage Genome in the Host Cell Genomes

In an extensive genomic analysis for virophage signatures in eukaryotic genomes [39,122], Blanc et al. screened more than 1000 genomes including those from protists, fungi, and basal metazoans. Four virophage core proteins families were used as signature genes, including a DNA-packaging ATPase, a cysteine protease, a MCP, and a mCP. Remarkably, 38 virophage-like elements were identified in the genome of *Bigelowiella natans*, a unicellular alga from the chlorarachniophytes group. The size and content of these endogenous elements were extremely heterogeneous and many of them corresponded to truncated genes, but six presumably complete copies flanked with terminal inverted repeats (TIRs) were described. The virophage-like copies identified exhibited a lower GC% compared to flanking host-sequences, which suggested that they had recently been integrated. Interestingly, most of the virophage-like elements were transcriptionally active, some being highly expressed including the morphogenesis elements and one integrase domain. The finding of Blanc et al. reinforced the scenario previously proposed by Fischer et al. of host cell–giant virus–virophage interaction [87]. Under this scenario, virophage integration represents an adaptive strategy advantageous for both virophage and host cell. On the one hand, endogenized virophage sequences confer to the cellular host population a protection from giant virus attacks. On the other hand, the integration of virophages may increase the frequency of their contact with a giant virus host.

Recently, Fischer et al. demonstrated that the Mavirus virophage can integrate at several loci in the nuclear genome of its cellular host *C. roenbergensis* [40]. This mechanism may be allowed by the presence of a retroviral integrase packaged in the virophage particle [117]. Three nuclear localization signals (NLSs) also encoded by Mavirus probably promote the translocation of the Mavirus genome–integrase complex to the nucleus of the host cell *C. roenbergensis* [117]. The retroviral integrase might then interact with the Mavirus genome to enable its integration into the host cell genome. Mavirus was found to remain latent until superinfection by its viral host CroV that triggers provirophage expression. This expression seems most likely to be mediated by a CroV-encoded late transcription factor [40,123]. This mechanism might be allowed by specific promoters shared by Mavirus and CroV [87]. Provirophage expression then leads to a massive production of Mavirus particles from the giant virus factory. Mavirus-produced particles do not seem to have a protective role for their host cell at this stage. This is probably because CroV infection causes cell lysis. However, this lysis releases virophage particles that can inhibit CroV replication in neighboring cells co-infected with Mavirus and CroV.

**Figure 5:**
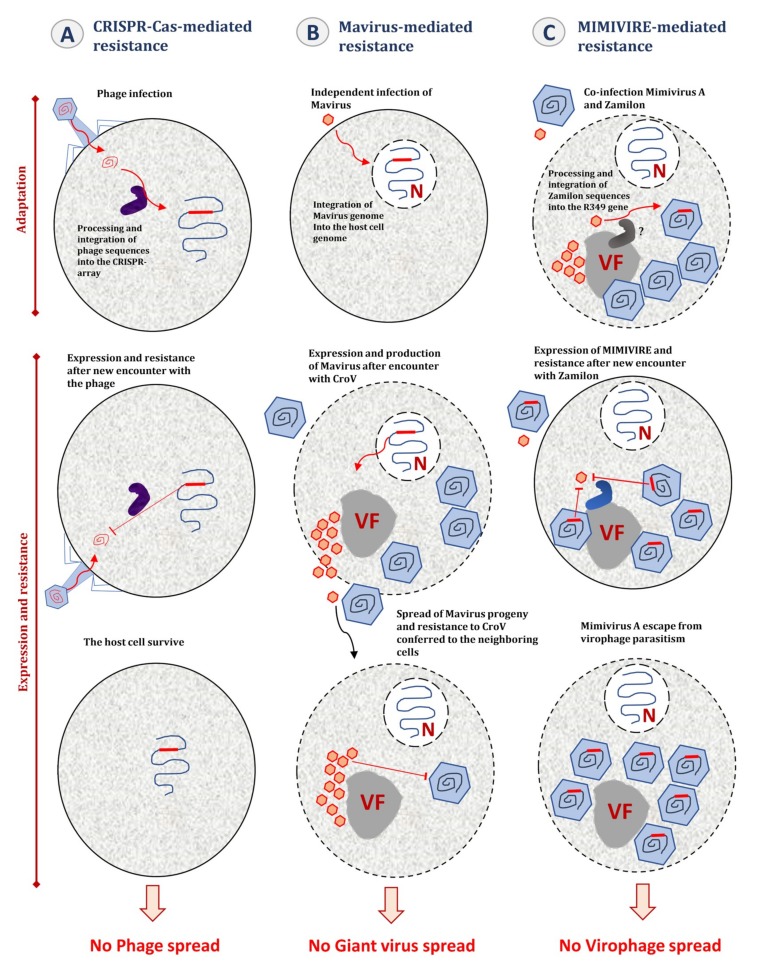
Analogies between the CRISPR-Cas-, the Mavirus- immunity, and the MIMIVIRE-mediated immunity systems. All these mechanisms seem to implicate a record of genetic sequences and their integration into the genome of immunized organisms at their first step (adaptation). (**A**) For the CRISPR-Cas system, the host cell incorporates DNA pieces from the invading phage (proto-spacers) into the CRISPR arrays (spacers) (not represented here). This mechanism is mediated by the Cas machinery (in purple). (**B**) After an independent entry of Mavirus in the host cell, the virophage integrates its genome into the host genome and becomes latent. This mechanism seems to involve Mavirus-encoded retroviral integrase. (**C**) Mimiviruses from lineage A have integrated DNA sequences from Zamilon into their R349 genes. However, unlike the CRISPR-Cas and Mavirus systems, the integration mechanism of Zamilon DNA has not been characterized. The next step of the resistance mechanism is marked by the expression of the integrated genetic pieces. (A) For the CRISPR-Cas system, transcripts of the spacers are used as a guide for the Cas proteins to cut foreign DNA after new encounters with the phage. This phenomenon allows the host cell to survive and prevents the spread of the phage. (B) Infection of the host cell with Cafeteria roenbergensis virus (CroV) activates the expression and production of Mavirus. The host cell is lysed at the end of the CroV replication cycle but spread of the virophage progeny confers resistance to neighboring cells, allowing their survival and avoiding CroV spread. (C) The expression of the R349 gene containing Zamilon DNA and of the Cas-like proteins (in blue) is associated with the inhibition of Zamilon replication, conferring to lineage A mimiviruses a resistance to this virophage.

Accordingly, it has been proposed that *C. roenbergensis* adopts an altruistic host-defense strategy mediated by the provirophage against the giant virus CroV [124]. This particular host defense mechanism has raised several questions regarding its potential analogy with CRISPR-Cas immunity in bacteria and archaea. In the model proposed by Koonin et al., both CRISPR-Cas system and provirophage host-defense mechanism involve the establishment in the cellular genome of an immunological memory of past infections (Figure 5) [124]. In the case of the CRISPR-Cas system, genome integration involves DNA fragments from the invading virus. In contrast, in the case of the provirophage-mediated mechanism, this is not the genetic material of the giant virus that is integrated into the cellular host it threatens, but that of a virus that is able to silence the giant virus replication and therefore to rescue the cellular population (Figure 5). Another point of analogy evocated between CRISPR-Cas immunity and the provirophage defense system is the fate of the cellular host after the encounter with its infectious agent. As described for the provirophage defense system, some types of CRISPR-Cas systems mediate an altruistic mode by triggering programmed cell death or dormancy to prevent virus propagation in the neighboring cells [125,126,127,128]. In addition, these two distinct mechanisms seem to evolve from self-synthesizing transposons, a class of mobile genetic elements. Indeed, the CRISPR-Cas system adaptation machinery has been proposed to have evolved from Casposons, self-replicating mobile genetic elements identified in various prokaryotic genomes [129]. Likewise, the Mavirus virophage shares a close evolutionary relationship with Polintons [109]. As for CRISPR-Cas systems, the provirophage mediated host-defense uses integration of sequences from the invading viruses, and it has therefore been suggested that this represents an adaptive immunity mechanism.

Such a mechanism of virus integration into the host cell genome has long been known for the adeno-associated virus (AAV), a single-stranded DNA dependoparvovirus that requires the presence of a helper virus (e.g., Adenovirus) to replicate [130,131]. In the absence of co-infection with its helper, AAV establishes a latent infection by specifically integrating itself into a preferential site of the host cell chromosome [132,133,134]. AAV integration seems to be widespread among the human population where up to 90% of adults are seropositive for the infection [135]. Subsequent infection of the latently infected cells with a helper virus can efficiently rescue the latent AAV genome and thus allows a fully permissive infection [136]. Moreover, once replicating, AAV is able to affect the replication of its helper by inducing a 50-fold decrease in the helper virus production and a maximum 40-fold reduction in its DNA synthesis [137]. Nevertheless, AAV autonomous replication has been reported for some human cells such as keratinocytes [138]. It has also been reported that genotoxic agents activate AAV replication even in the absence of its helper [139]. Such events have never been described for Mavirus or any other virophage.

Although little is known about virophage genomes endogenized in host cell chromosomes, this mechanism does not seem very widespread in amoebae. In a recent investigation conducted by Chelkha et al., no virophage like-sequences were detected in the genomes of sixteen *Acanthamoeba* spp. [140]. As described above, it has been proposed that, unlike Mavirus, *Acanthamoeba* virophages infect their host cells simultaneously with the giant virus. Therefore, vertical transmission of provirophage could be prevented by cell lysis.

## 7. MIMIVIRE, a Giant Virus Defense System

To repress virophage attacks, it has been proposed that some giant viruses have evolved a defense mechanism that, as CRISPR–Cas systems, involves the collection of genetic information from virophages and their use for specific inhibition of virophage multiplication (Figure 5). Indeed, Levasseur et al. demonstrated that mimiviruses of lineage A acquired an adaptive immunity against Zamilon virophage through the incorporation in their genomes of short Zamilon sequences [41,141]. A 28 nucleotide-long Zamilon sequence was indeed found in all genomes from lineage A virus, and a 15 nucleotide-long fragment of it is repeated four times exclusively in the genomes from lineage A mimiviruses whereas not in those from lineages B and C. Moreover, two potential Cas-like genes that encode a helicase and a nuclease (R350 and R354, respectively) were identified near the gene (R349) that contains this repeat. This operon of three genes was designated the MIMIVIRE system for MIMIvirus VIrophage Resistance Element. The involvement of each gene in the resistance mechanism has been validated by silencing experiments [41]. In addition, the structural and functional characterization of the MIMIVIRE nuclease suggests that it is a Cas-4 homolog [142], and the knock-out of the R349 gene by homologous recombination has restored the permissiveness of Mimivirus A to Zamilon [143]. Furthermore, a recently isolated Mimivirus of lineage A that has a MIMIVIRE sequence containing a single repeat instead of 4 virophage repeats, is susceptible to Zamilon infection [143]. However, the existence in giant viruses of such a mechanism of immunity based on the integration of invader’s DNA has been criticized. Thus, several issues were raised about the analogy between MIMIVIRE and CRISPR-Cas systems [144]. The first issue is the absence of sharp demonstration of the self/non-self-discrimination process in MIMIVIRE. In the CRISPR-Cas system, such a mechanism is mediated by the protospacer adjacent motif (PAM) that allows the defense system to distinguish self from non-self-DNA [145,146,147,148]. Second, Zamilon repeats are not regularly interspaced with a conserved DNA sequence and the genomic environment of R349 orthologs differs considerably in mimiviruses of lineage A and of lineages B or C. An alternative scenario to an adaptive defense system based on specific nucleic acid sequences has been proposed, consisting of protein–protein interference [144,149]. Nevertheless, to date, this model has not been the subject of any experimental demonstration.

## 8. Virophages, a Source of Controversy

Over the past decade, the classification of virophages has been the subject of intense debate within the scientific community [150]. Krupovic et al. argued that virophages should be classified within the group of classical satellite viruses [151,152]. According to the International Committee on Taxonomy of Viruses (ICTV) definition, satellite viruses are “subviral agents lacking genes that could encode functions needed for replication and depending on the coinfection of a host cell with a helper virus for their multiplication”. Unlike virophages, the description of satellite viruses goes back several decades with the discovery of the satellite tobacco necrosis virus (STNV) [8,153,154]. SNTV is a small virus (18 nm in diameter) with a single-stranded RNA genome of 1.2 kb that cannot replicate in the absence of its helper virus, the tobacco necrosis virus (TNV), a plant pathogenic RNA virus from the genus *Necrovirus* [155,156]. The second such relationship was described in 1966 with the discovery of the adeno-associated satellite virus (AAV) and its helper adenovirus [157]. AAV has a single-stranded DNA genome of approximately 5 kb and an enveloped capsid that is 20 nm in diameter [130,158]. AAV has been shown to replicate with other helper viruses, including herpes simplex virus, cytomegalovirus, human papillomavirus, and more recently human bocavirus [159,160,161,162]. Subsequently, other satellite viruses with various genome types (ssRNA or ssDNA) were discovered, which have hosts of different origins, including arthropods, animals, and plants [105]. Most of them seem unable to replicate in their host cells without the assistance of a specific helper virus.

Krupovic et al. suggested that virophages are no more than a subgroup of satellite viruses with a double-stranded DNA genome [151,152]. They stated several concerns regarding what has been considered as specific to virophages but not to satellite viruses, namely the intracellular localization, the genome expression, and the effect on the host/helper virus. Indeed, the fact that virophages are produced from the same virus factory as their host viruses was not considered as sufficient to deserve a specific classification. Previous studies have shown that satellite viruses follow their helper viruses wherever they go. STNV replication occurs in the cytoplasm of the host cells near its helper TNV, while both AAV and its helper adenovirus replicate in the nucleus.

The second point of controversy was the specific polyadenylation signals shared between the transcripts of some giant viruses and their virophages. The same is true for some satellite viruses that share specific RNA signals with their helpers. This feature was considered to show the dependence on the transcription–replication complex of the helper/host virus, without supporting the virophage concept.

The negative effect of virophages on their viral hosts is probably the strongest support of the virophage concept. However, it has been observed that even satellite viruses could affect the replication of their helpers. Indeed, infection with STNV could reduce the titer of TNV to undetectable levels, resulting in fewer and smaller necrotic lesions than with infection with TNV alone. In addition, co-infection with AAV reduces the production of adenovirus particles by up to 100-fold and the replication of adenovirus DNA by up to 10-fold [163,164,165]. This suggests that the negative effect of a virus on its helper virus may be not specific to virophages.

**Figure 6:**
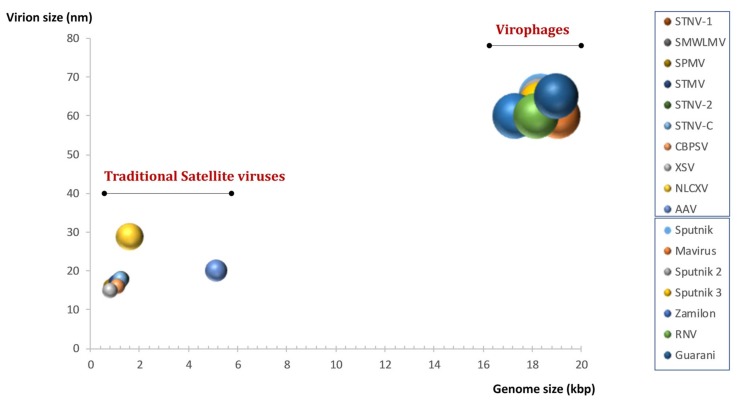
The parasites of the giants are giants. Plot comparing the virion and genome sizes for known virophages and some traditional satellite viruses. The ball sizes are proportional to the capsid sizes.

Under the light of previous data, the ninth report of the International Committee on Taxonomy of Viruses for Virus Taxonomy (ICTV, 8 November 2018), classified virophages in the group of satellite viruses. However, this classification has been criticized on the basis of two major points that question the classification of virophages as subviral agents [31,166]. First, virophages are fully functional viruses, whose genomes encode their own structural and DNA replication proteins. The size, the nature, and the complexity of their genomes make them closer to autonomous *bona fide* viruses than to subviral agents or defective particles (Figure 6). Furthermore, virophages fits the current definition of a virus as “a capsid-encoding organism that is composed of proteins and nucleic acids, self-assembles in a nucleocapsid and uses a ribosome-encoding organism for the completion of its life cycle” [32]. This is untrue for many subviral agents. In addition, MCPs of the different virophages seem related to each other, while they are not present in other known viruses (including those from the bacteriophage PRD1-adenovirus lineage). This strongly supports the uniqueness of virophages as new viral entity.

**Figure 7:**
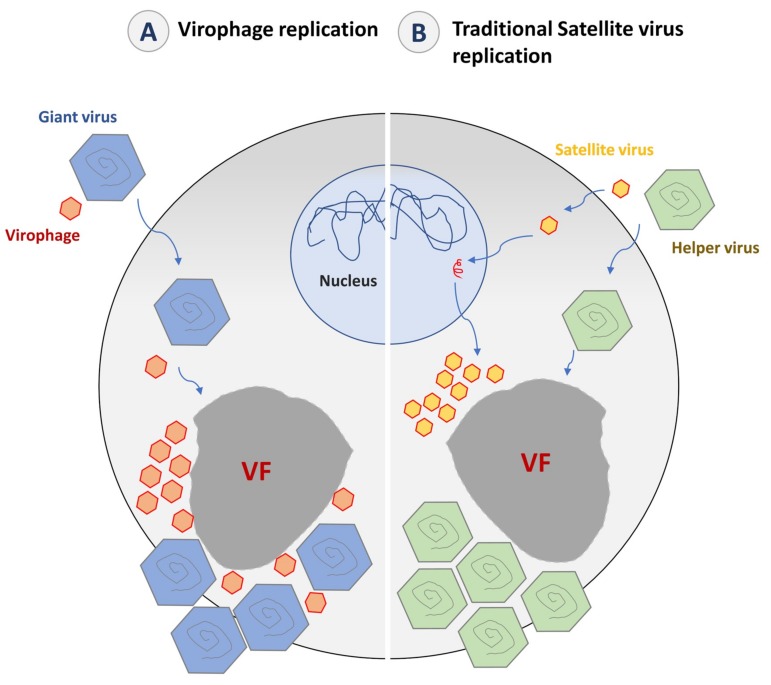
Virophages and satellite virus’ lifestyle. (**A**) The replication of virophages is supposed to occur entirely in the virus factory of its giant virus host, depending of the giant virus expression/replication complex. (**B**) The concept of satellite virus implicates that the virus initiates the expression and replication of its genome in the nucleus using the host cell machinery and then goes to the cytoplasm. In the cytoplasm, the satellite virus hijacks the morphogenesis machinery of its helper virus to produce its progeny.

The second point raised by researchers defending the virophage concept is that virophages are viruses that really infect other viruses (Figure 7). Indeed, it has been proposed that the genes of virophages be expressed by the transcription mechanisms of giant viruses. This is suggested by the mimiviral polyadenylation signals and late promoter motifs found upstream of 12 Sputnik genes and all Mavirus genes. In addition, the negative effect of virophages is different from that observed with some satellite viruses as for the case of STNV. Thus, in some cases, infection by this satellite virus can increase the infectivity of its helper TNV. Such an effect has never been observed with virophages. Moreover, unlike satellite viruses, some virophages are able to increase the production rate of abnormal particles [30].

## 9. Definition and Classification

Great debates in science usually end with a compromise solution and the question of the existence of the virophage concept is no exception. In 2015, Fischer and Krupovic proposed to redress the classification of satellite viruses, previously classified as subviral agents, by the creation of two new viral families and seven new genera [105]. The family *Sarthroviridae* and five genera have been proposed to allocate satellite viruses of plants and insects. As for virophages, the creation of the family *Lavidaviridae* (Large virus-dependent or -associated virus), which includes two genera (*Sputnikvirus* and *Mavirus*), has been proposed. This taxonomic proposal has been approved by the 10th report of the ICTV, which now considers virophages as a new divergent family of double-stranded DNA satellite viruses infecting protists that are not subviral agents. Recent discoveries related to virophages, including the MIMIVIRE system and the Mavirus mediated host-defense, support the uniqueness of virophages and strengthen the concept of “a virus infecting another virus” [31,166]. To our knowledge, such mechanisms have never been described for satellite viruses. Today, an increasing number of publications use the term virophage to designate small *bona fide* viruses “with the distinguishing feature that the organisms they infect are viral themselves” [31,166].

## 10. Conclusions

Over the last decade, virophages have emerged as fascinating viruses that require the presence of a giant virus co-infecting the host cell to replicate. While negative consequences of virophage integration into the host cell genome has recently been discussed [123], they do not seem to cause cytopathic effect in their host cell alone. Their discovery has significantly contributed to expand our knowledge regarding the diversity, evolution, and complexity of viruses. Virophages have been defined as the first viral parasites to infect another viral entity. Furthermore, they have been involved in two varieties of host defense mechanisms, the Mavirus-mediated immunity that protects protists from the giant CroV and the MIMIVIRE system that confers resistance to mimiviruses from virophages. Research in the virophage field is in its initial phase and further discoveries that could challenge the current concept of a virus may be revealed in the coming years.

## Figures and Tables

**Table 1:** Virophages discovered by co-culture and genomic and their main features.

Virophage	Year of Description	Place of Isolation	Associated Giant Virus	Capsid Size (nm)	Genome Features			GenBank/EBI Accession No.	References
					Size (bp)	Number of ORFs	G+C (%)		
Sputnik	2008	France	Mamavirus	50–74	18,343	21	27	EU606015	[30]
Mavirus	2010	USA	Cafeteria roenbergensis virus	60	19,063	20	30	NC_015230	[87]
Sputnik 2	2012	France	Lentillevirus	50–74	18,338	20	27	NC_023846	[38]
Sputnik 3	2013	France	APMV ^1^	50–74	18,338	20	27	NC_023847	[88]
PgVV	2013	North Sea	Phaeocystis globosa virus	N.d.	19,527	16	36	NC_021333	[62]
Zamilon	2014	Tunisia	Mont1	60	17,276	20	30	NC_022990	[89]
RNV	2014	Brazil	Sambavirus	50–70	18,145	20	27	MG676470	[90]
Platanovirus saccamoebae virophage	2018	Germany	KSL5 virus	50	N.d.	N.d.	N.d.	N.d.	[91]
Guarani	2019	Brazil	APMV ^1^	50–74	18,967	22	26	LS999520	[92]
3 CpV-PLV virophages	2019	USA	CpV-BQ2	N.d.	21,750- 22,879	19–23	30–39	MH920636/ MH919296/ MH919297	[69]

PgVV: Phaeocystis globosa Virus Virophage RNV: Rio Negro Virophage. CpV-PLV: Chrysochromulina parva Virus - Polintons-like virophages, N.d.: Not determined. ^1^ this giant virus was only used as helper to isolate the virophage.

**Table 2:** Virophages discovered by metagenomics to date and their main features.

Virophage	Year of Description	Origin of the Metagenome	Associated Giant Virus	Genome Features			Genbenk/EBI Accession No.	References
				Size (bp)	Number of ORFs	G+C (%)		
OLV	2011	Antarctica	Organic lake phycodnavirus	26,421	24	37	HQ704801	[55]
YSLV1	2013	USA	N.d.	27,849	26	33	KC556924	[100]
YLSV2	2013	USA	N.d.	23,184	21	34	KC556925	[100]
YLSV3	2013	USA	N.d.	27,05	23	35	KC556926	[100]
YLSV4	2013	USA	N.d.	28,306	34	37	KC556922	[100]
ALM	2013	Antarctica	N.d.	17,767	22	27	KC556923	[100]
YLSV5	2014	USA	N.d.	29,767	32	51	KM502589	[101]
YLSV6	2014	USA	N.d.	24,837	29	27	KM502590	[101]
YLSV7	2014	USA	N.d.	23,193	26	27	KM502591	[101]
3 RVPs (incompletes)	2015	USA	*Mimiviridae*	Up to 26, 209	Up to 21	-	-	[102]
Zamilon 2 (incomplete)	2015	USA	*Mimiviridae*	6716	15	32	-	[99]
QLV	2016	China	N.d.	23,379	25	33	KJ854379	[104]
DSLV1	2016	China	N.d.	28,788	28	43	KT894027	[103]
17 Freshwater virophages (10 incompletes)	2017	USA	*Mimiviridae*	13,800–25,800	13–25	-	-	[98]

OLV: Organic Lake virophage. YSLV: Yellowstone Lake virophage, ALM: Ace Lake Mavirus, RVPS: Rumen metagenome virophages, QLV: Qinghai Lake virophage, DSLV1: Dishui Lake virophage, N.d.: Not determined.
